# Acknowledgement to Reviewers of *Nanomaterials* in 2013

**DOI:** 10.3390/nano4010155

**Published:** 2014-02-27

**Authors:** Nanomaterials Editorial Office

**Affiliations:** MDPI AG, Klybeckstrasse 64, CH-4057 Basel, Switzerland; E-Mail: nanomaterials@mdpi.com

The editors of *Nanomaterials* would like to express their sincere gratitude to the following reviewers for assessing manuscripts in 2013:

Ackland, K.
Advincula, Rigoberto C.
Al-Haik, Marwan
Albdiry, M. T.
Arancibia-Miranda, Nicolas
Bae, Joonwon
Baptista, Pedro V.
Binions, Russell
Bouchiat, Vincent
Brendlé, Jocelyne
Breza, Juraj
Capobianco, John A.
Catledge, S. A.
Cavaleiro, Albano
Chang, Chi-Jung
Chang, Haixin
Chen, Guanying
Chen, Jem-Kun
Chen, Kuei-Hsien
Chen, Zhan
Chi, Kai-Ming
Christesen, Steven
Cobas, Enrique
Colaco, Rogerio
D’Urso, L.
Decoster, Mark
Dickerson, James H.
Dishari, Shudipto Konika
Dixon, David A.
Fan, Xin
Fan, Yue
Garrone, Edoardo
Gasparotto, Alberto
Gu, Yizhuo
Guicheteau, Jason
Guo, Shouwu
Hao, Qingli
Hasegawa, George
Hernandez-Cordero, Juan
Hill, Matthew R.
Hirsch, Thomas
Hong, Minghui
Huang, Xinyu
Johansson, Christer
Kah, Melanie
Kalantar-Zadeh, Kourosh
Kang, Sang Wook
Kang, Zhenhui
Katti, Kalpana S.
Kawasaki, Hideya
Kim, Dong-Eun
Kim, Jong-Duk
Knaus, Simone
Kober, Mariana
Komura, Kenichi
Krüger, Michael
Laborie, Marie-Pierre
Lagugne-Labarthet, Francois
Lazzara, Giuseppe
Levitsky, Igor
Li, Hao
Liu, Juewen
Liu, Xiaogang
Lockard, Jenny
Long, Yi
Ma, Pengcheng
Mączka, Mirosław
Maffucci, Antonio
Mai, Liqiang
Malmström, Eva
Marquette, Christophe
Martinez-Boubeta, C.
Marty, Laëtitia
Matsumoto, Yasumichi
Morell, Gerardo
Mougin, Karine
Müller, Alejandro J.
Muschol, Martin
Nair, Sankar
Tobias, Gerard
Nersisyan, H. H.
O’Reilly, A. J.
Parkinson, Bruce A.
Pedroni, Marco
Peng, Tianyou
Pereña, Jose Manuel
Pestov, Dmitry
Pimentel-Dominguez, R.
Pyatenko, Alexander
Ragauskas, Arthur J.
Richard, Cyrille
Rodrigues, Debora F.
Roh, Kwang Chul
Rosseeva, Elena
Rossi, M.
Seferos, Dwight S.
Shalav, Avi
Shin, Kwanwoo
Siebbeles, Laurens D. A.
Sierin, Lim
Singh, Virendra
Song, Changsik
Stitzel, Shannon
Suh, Yung Doug
Takenobu, Taishi
Nann, Thomas
Tomar, Vikas
Torsi, Luisa
Umar, Akrajas Ali
Wang, Xiangke
Wang, Xin
Wang, Yuansheng
Wang, Zhongchang
Weimer, Jeffrey
Wlodarski, Wojciech
Yamada, Toshishige
Yan, Chenglin
Zhang, Xiao
Zheng, Wenjun

